# Molecular biology of autoinflammatory diseases

**DOI:** 10.1186/s41232-021-00181-8

**Published:** 2021-10-11

**Authors:** Junya Masumoto, Wei Zhou, Shinnosuke Morikawa, Sho Hosokawa, Haruka Taguchi, Toshihiro Yamamoto, Mie Kurata, Naoe Kaneko

**Affiliations:** Department of Pathology, Ehime University Graduate School of Medicine and Proteo-Science Center, Shitsukawa 454, Toon, Ehime 791-0295 Japan

**Keywords:** Interleukin-1, NF-κB, Type I interferon, Autoinflammatory diseases

## Abstract

The long battle between humans and various physical, chemical, and biological insults that cause cell injury (e.g., products of tissue damage, metabolites, and/or infections) have led to the evolution of various adaptive responses. These responses are triggered by recognition of damage-associated molecular patterns (DAMPs) and/or pathogen-associated molecular patterns (PAMPs), usually by cells of the innate immune system. DAMPs and PAMPs are recognized by pattern recognition receptors (PRRs) expressed by innate immune cells; this recognition triggers inflammation. Autoinflammatory diseases are strongly associated with dysregulation of PRR interactomes, which include inflammasomes, NF-κB-activating signalosomes, type I interferon-inducing signalosomes, and immuno-proteasome; disruptions of regulation of these interactomes leads to inflammasomopathies, relopathies, interferonopathies, and proteasome-associated autoinflammatory syndromes, respectively. In this review, we discuss the currently accepted molecular mechanisms underlying several autoinflammatory diseases.

## Background

**Fig. 1:**
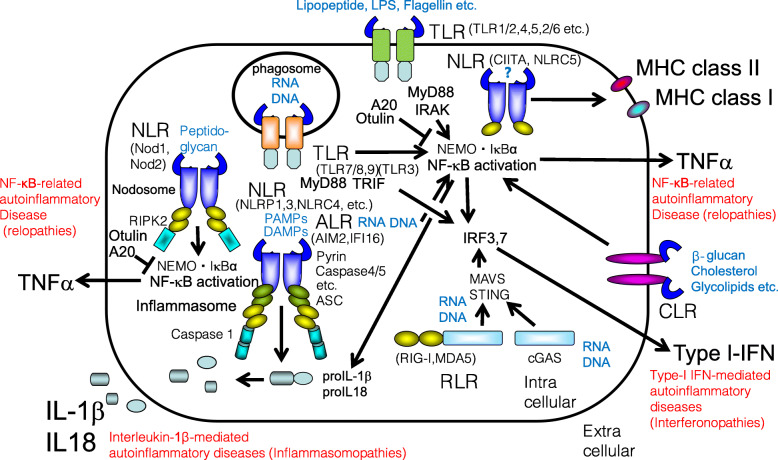
Pattern-recognition receptors in innate immune cells. PRRs include Toll-like receptors (TLRs), Nod-like receptors (NLRs), and RIG-I-like receptors (RLRs) that recognize various damage-associated molecular patterns (DAMPs) and pathogen-associated molecular patterns (PAMPs) to initiate immune responses. These receptors are also called innate immune receptors. Gain-of-function mutations of the innate immune receptors or loss-of-function mutations of their inhibitors are related to autoinflammatory diseases. Red characters indicate DAMPs and PAMPs. Red characters indicate classified autoinflammatory diseases

The human body has evolved various adaptive responses that protect against cell and tissue damage caused by physical, chemical, and biological factors. Such factors include molecules released by damaged tissues, metabolites, and/or infection (e.g., by bacteria, viruses, and parasites) [1–4]. Inflammation, an adaptive response to cell injury, generates damage-associated molecular patterns (DAMPs) and/or pathogen-associated molecular patterns (PAMPs), which are then recognized by pattern recognition receptors (PRRs) expressed mainly by innate immune cells [5]. PRRs include Toll-like receptors (TLRs), Nod-like receptors (NLRs), C-type lectin receptors (CLRs), and RIG-I-like receptors (RLRs) that recognize DAMPs and PAMPs to initiate immune responses. These receptors are also called innate immune receptors [6] (Fig. 1).

Autoinflammatory diseases are strongly associated with dysregulation of these PRR-containing interactomes, which include inflammasomes, nuclear factor (NF)-κB-activating signalosomes, type I interferon-inducing signalosomes, and immuno-proteasomes; dysfunction of these interactomes results in inflammasomopathies, relopathies, interferonopathies, and proteasome-associated autoinflammatory syndromes (PRAAS), respectively [7–11]. This explains the pathogenesis of autoinflammatory diseases involving recurrent inflammatory flare-ups in the absence of autoantibodies or antigen-specific T lymphocytes [12]. Knowledge of the molecular mechanism(s) underlying the functions of these innate immune receptors is useful for the treatment and management of individuals with autoinflammatory diseases (Fig. 1).

## Interleukin-1β-mediated autoinflammatory diseases (inflammasomopathies)

**Fig. 2:**
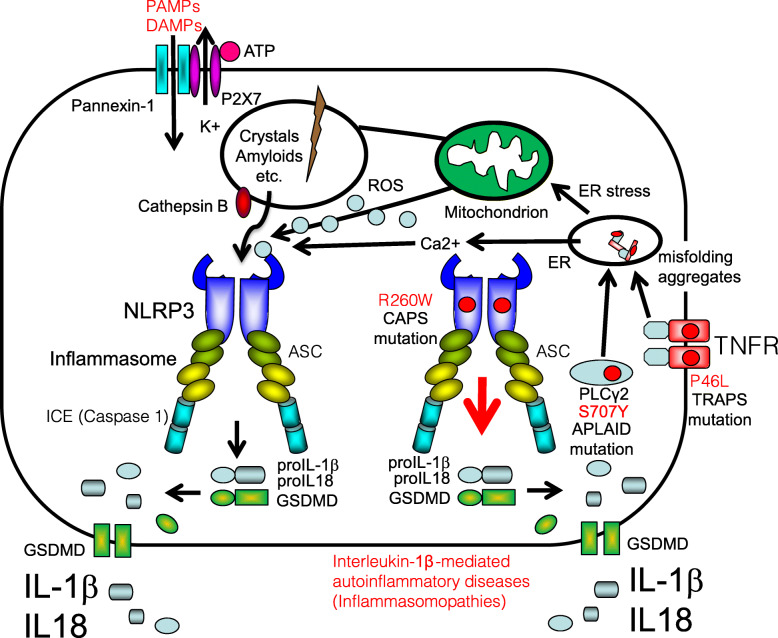
Cryopyrin-associated periodic fever syndrome (CAPS), TNF receptor-associated periodic syndrome (TRAPS), and autoinflammation and phospholipase Cγ2 (PLCγ2)-associated antibody deficiency and immune dysregulation (APLAID) are related to NLRP3 inflammasome. Gain-of-function mutations of NLRP3 (e.g., R260W) leads to prolonged activation of NLRP3 inflammasome. Autoinflammatory syndrome caused by the gain of function of NLRP1, NLRP12, or other NLRP mutations is thought to be basically caused by the same mechanisms. Mutated TNFRSF1A (TNFR) in patients with TRAPS is misfolded and accumulated in the endoplasmic reticulum (ER), causing ER stress and increased generation of mitochondrial reactive oxygen species (ROS) that activates the NLRP3 inflammasome. PLCγ2 mutation in patients with APLAID (e.g., S707Y) leads to calcium influx from the ER and increased cytoplasmic Ca2+ levels promote activation of NLRP3 inflammasome

When NOD-like receptors harboring a PYRIN domain (PYD) (e.g., NLRP1, NLRP2, NLRP3, NLRP6, NLRP9, and NLRP12) and other pyrin domain-containing PRRs (e.g., pyrin, AIM2, and IFI-16) sense DAMPs, PAMPs, or intracellular microenvironmental changes (e.g., potassium efflux), they interact with an adaptor protein apoptosis-associated speck-like protein containing a caspase-recruitment domain (ASC) via PYD, and pro-caspase-1 via a caspase-recruitment domain (CARD). This interaction activates caspase-1, a process accompanied by pyroptotic cell death [13–29]. NOD-like receptors carrying a CARD domain or CARD including proteins alternatively interact with caspase-1 via CARD with ASC and pro-caspase-1 such as NLRP1, NLRC4, CARD8, and caspase-11 [30–32]. The resulting complexes act as a sensor of cell injury; this sensor is referred to as the inflammasome, an interleukin (IL)-1β- and IL-18-processing platform that plays a crucial role in the maturation and secretion of these cytokines from cells. The process is accompanied by a type of cell death, named pyroptosis, which is triggered by cleavage of gasdermin D (GSDMD) [33, 34] (Fig. 2). Below, we discuss specific inflammasomopathies.

### Cryopyrin-associated periodic syndrome

Cryopyrin is the same protein as NLRP3 which was named by the nomenclature committee. Gain-of-function mutations in NLRP3 lead to cryopyrin-associated periodic syndrome (CAPS), a spectrum of diseases that includes familial cold autoinflammatory syndrome (FCAS, formerly termed familial cold urticaria (FCU)), Muckle–Wells syndrome (MWS), and neonatal-onset multisystem inflammatory disease (NOMID; also called chronic infantile neurologic cutaneous and articular syndrome (CINCA)). Currently, 248 variants of the *CIAS1* gene have been reported by “INFEVERS” (https://infevers.umai-montpellier.fr/web/search.php?n=4) [35]. The NLRP3 mutations in CAPS result in constitutive activation of the NLRP3 inflammasome (i.e., the threshold for stimulation is extremely low). Activation of the inflammasome leads to excess pyroptosis of cells expressing components of the NLRP3 inflammasome; these cells secrete excessive amounts of activated IL-1β upon autoinflammatory attack [36–41] (Fig. 2). Corresponding common diseases caused by the similar signaling are shown in Table 1.

**Table 1:** The corresponding diseases caused by the similar signaling of autoinflammatory diseases

Type of autoinflammatory diseases	Responding proteins	Functions	Corresponding diseases with the similar signaling
**Inflammasomopathies**			
CAPS	Cryopyrin/NLRP3	PRR for ROS, K^+^ efflux, cathepsin B detection	Metabolic syndrome:
			Gout [42]
			Atherosclerosis [43]
			Type 2 diabetes [44]
			Neurodegenerative disease:
			Alzheimer’s disease [45, 46]
			Parkinson’s disease [47]
			Amyotrophic lateral sclerosis [48]
			Multiple sclerosis [49]
			Infections and aberrant inflammatory responses:
			Septic shock syndrome [50, 51]
			Ischemic diseases:
			Myocardial infarction [52]
			Stroke [53]
NAIAD	NLRP1	PRR against *Anthrax* toxin detection	Infections and aberrant inflammatory responses:
			*Anthrax* lethal toxin [54]
			Neurodegenerative disease:
			Alzheimer’s disease [46]
			Ischemic diseases:
			Stroke [53]
NLRP12-AD	NLRP12	PRR for *Yersinia pestis* detection	Infections and aberrant inflammatory responses:
			*Yersinia pestis* [55]
			*Plasmodium chabaudi* [56]
		NF-κB inhibition	Regulation of inflammation:
			*Salmonella typhimurium* [57]
			*Brucella abortus* [58]
TRAPS	TNFRSF1A	TNF receptor	Infections and aberrant inflammatory responses:
			Tumor necrosis factor [59]
APLAID	PLCγ2	Cleavage PIP to DAG	Immunodeficiency:
			Common variable immunodeficiency [60]
			PLCγ2-associated antibody deficiency and immune dysregulation syndrome (PLAID) [60]
			Familial cold autoinflammatory syndrome 3 [60]
			Neurodegenerative disease:
			Alzheimer’s disease [61, 62]
			Lewy body dementia [62]
			Frontotemporal dementia [62]
FMF	Pyrin	Virulence sensor	Infections and aberrant inflammatory responses:
			*Yersinia pestis* infection [63]
PFIT	WDR1	Actin assembly, leukocyte migration	Infections and aberrant inflammatory responses:
			*Listeria monocytogenes* dissemination [64]
PAAND	Pyrin	Virulence sensor	Infections and aberrant inflammatory responses:
			*Yersinia pestis* infection [63]
PAPA syndrome	PSTPIP1/CD2BP1	Pyrin regulation	Immunodeficiency:
			Common variable immunodeficiency [65]
MKD	MVK	Lipid metabolism	Metabolic syndrome:
			Atherosclerosis [66]
NLRC4 inflammasomopathies	NLRC4	PRR for flagellin detection	Infections and aberrant inflammatory responses:
			*Pseudomonas aeruginosa* infection [67]
			Macrophage activation syndrome (MAS) [68]
**Relopathies**			
BS/EOS	NOD2	PRR for MDP	Infections and aberrant inflammatory responses:
			*Mycobacterium tuberculosis* infection [69]
HA20	A20/TNFAIP3A	Deubiquitinating for NF-κB regulation	Infections and aberrant inflammatory responses:
			Systemic lupus erythematosus (SLE) [70]
			Rheumatoid arthritis [71]
IAALUCD	LUBAC	Ubiquitinating for NF-κB regulation	Infections and aberrant inflammatory responses:
	HOIL-1/ RBCK1		*Salmonella enterica* infection [72]
	HOIP/RNF31		*Legionella pneumophila* infection [72]
	SHARPIN		*Shigella flexneri* infection [72]
ORAS	OTULIN	Deubiquitinating for NF-κB regulation	Infections and aberrant inflammatory responses:
			*Salmonella Typhimurium* infection [73]
**IL-1 receptor-related autoinflammatory diseases:**			
DIRA	IL1RN	IL-1 receptor inhibitor	Infections and aberrant inflammatory responses:
			Inflammasomopathies [74]
**Interferonopathies**			
AGS	RNASEH2	RNase activity against viral RNA	Infections and aberrant inflammatory responses:
			SLE and other autoimmune diseases [75, 76]
			Cervical cancer via human papilloma virus [77]
	SAMHD1	dNTPase activity against viral RNA/DNA	Epstein-Barr virus infection [78]
			Human immunodeficiency virus infection [79]
	ADAR1	Viral RNA processing	Hepatitis B virus infection [80]
			Marburg and Ebola virus [81]
	MDA5/IFIH1	PRR for viral RNA detection	Paramyxovirus infection [82]
			Picornavirus infection [83]
SAVI	STING/TMEM173	PRR for viral DNA/RNA detection	Infections and aberrant inflammatory responses:
			SLE and other autoimmune diseases [76]
			ANCA-associated vasculitis [84]
			Herpes simplex virus infection [85]
COPA syndrome	αCOP	Transport vesicles between Golgi to ER	Infections and aberrant inflammatory responses:
			Interstitial lung disease [86]
			Capillaritis [87]
PRAAS/NNS/CANDLE	PSMB3,4,8,9	Proteasome for antigen processing	Infections and aberrant inflammatory responses:
			SLE and other autoimmune disease [87, 88]
			Cytomegalovirus infection [89]
			Hepatitis B virus infection [90]
			Influenza virus infection [91]
	POMP	Proteasome chaperone	Infections and aberrant inflammatory responses:
			Psoriasis [92]
			Human papilloma virus infection [93]
SMS	MDA5/IFIH1	PRR for viral RNA detection	Infections and aberrant inflammatory responses:
			SLE and other autoimmune diseases [75, 76, 94]
			Paramyxovirus infection [82]
			Picornavirus infection [83]
	RIG-I	PRR for viral RNA detection	Infections and aberrant inflammatory responses:
			SLE and other autoimmune diseases [95]
			Paramyxovirus infection [83]

### NLRP1-associated autoinflammation with arthritis and dyskeratosis

The NLRP1 inflammasome was the first “inflammasome” to be identified [14]. NLRP1 interacts with ASC through its PYD domain. ASC then interacts with pro-caspase-1 via its CARD domain, resulting in activation of IL-1β secretion; also, NLRP1 interacts with caspase-1 through its CARD domain to activate IL-1β secretion [96]. Currently, several mutations (A54T, A59P, A66V, M77T, R726W, T755N, F787_R843del, and P1214R) in the gene encoding NLRP1 have been identified (https://infevers.umai-montpellier.fr/web/search.php?n=31). Patients harboring these mutations exhibit dyskeratosis, oligo/polyarthritis, and recurrent fever, along with immunological dysfunction and vitamin A deficiency [97–99]. The mutations may trigger proteasome-dependent functional degradation of NLRP1, and degraded CARD-FIIND-containing-NLRP1 fragments act as a scaffold like ASC for inflammasome activation [100] (Fig. 2). Corresponding common diseases caused by the similar signaling are shown in Table 1.

### NLRP12 autoinflammatory syndrome

NLRP12 inhibits the activation of NF-κB. Mutations in NLRP12 are found in patients with hereditary periodic fever syndrome, the clinical signs of which are consistent with a diagnosis of CAPS [101]. Currently, 79 variants of the gene encoding NLRP12 have been reported (https://infevers.umai-montpellier.fr/web/search.php?n=9). Since some patients with gain-of-function mutations in NLRP12 exhibit symptoms similar to those of CAPS, the disease was named FCAS2 [102] and patients with NALP12 periodic fever syndrome respond to canakinumab (an anti-human IL-1β monoclonal antibody) and/or etanercept (a tumor necrosis factor (TNF) receptor-IgG heavy chain chimeric protein that acts as a bivalent antagonist of TNF activity) [102], the pathogenesis of NLRP12 autoinflammatory syndrome (NLRP12-AD) may explain the gain of function of the NLRP12 inflammasome by a similar mechanism of the NLRP3 inflammasome (Fig. 2). Corresponding common diseases caused by the similar signaling are shown in Table 1.

### TNF receptor-associated periodic fever syndrome

The causative gene product of TNF receptor-associated periodic fever syndrome (TRAPS) is TNF receptor superfamily member 1A (TNFRSF1A) [12]. So far, 180 variations of the TNFRSF1A gene have been reported (https://infevers.umai-montpellier.fr/web/search.php?n=2). The cysteine-to-cysteine disulfide bonds in the extracellular domain of TNFRSF1A for ER stress are thought to be important for disease pathogenesis. More than one-third of patients with TRAPS harbor the R92Q and P46L mutations [103]. In TRAPS, misfolding of mutated TNFRSF1A leads to accumulation of the protein in the endoplasmic reticulum (ER), which causes ER stress and increased generation of mitochondrial reactive oxygen species; this in turn activates inflammasomes [104, 105] (Fig. 2). Corresponding common diseases caused by the similar signaling are shown in Table 1.

### Autoinflammation and phospholipase Cγ2-associated antibody deficiency and immune dysregulation

Autoinflammation and phospholipase Cγ2 (PLCγ2)-associated antibody deficiency and immune dysregulation (APLAID) responds to PLCγ2 which encodes for a constitutively repressed phospholipase. The S707Y PLCγ2 mutation disrupts the autoinhibition of PLCγ2, thereby increasing PLCγ2 activity and calcium influx from the ER in the leukocytes of patients with APLAID [106, 107]. Increased cytoplasmic Ca2+ levels promote the assembly of the NLRP3 inflammasome [108] (Fig. 2). Corresponding common diseases caused by the similar signaling are shown in Table 1.

### Familial Mediterranean fever

**Fig. 3:**
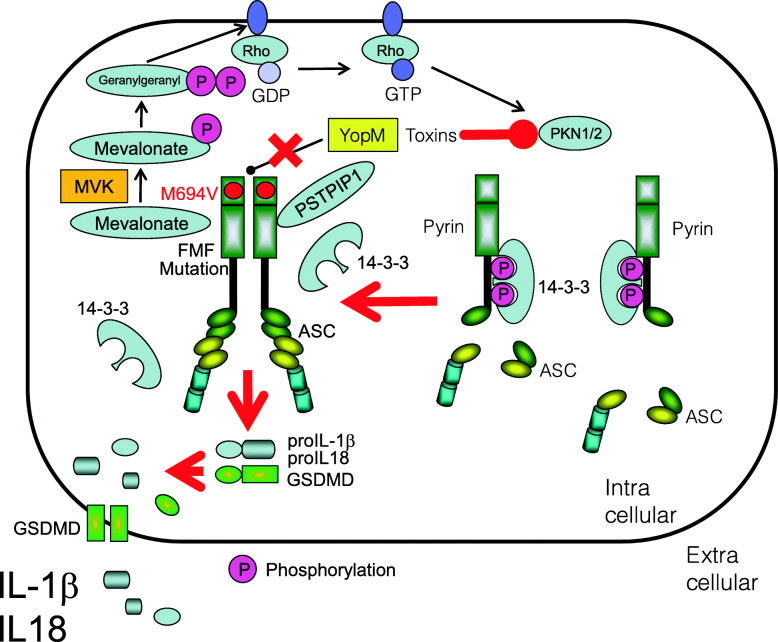
Prolonged activation of pyrin inflammasome in patients with familial Mediterranean fever (FMF). Upon *Yersinia pestis*-like bacterial infection, *Yersinia* outer protein (Yop)M interacts with pyrin to inhibit pyrin inflammasome to avoid further anti-bacterial responses. In patients with FMF, pyrin-harboring mutant human B30.2 domains enable to interact with YopM, resulting in prolonged pyrin inflammasome activation and IL-1β secretion

The causative gene of familial Mediterranean fever (FMF), *MEFV*, encodes pyrin (also named marenostrin) [109, 110]. Currently, 389 variants of *MEFV* have been reported (https://infevers.umai-montpellier.fr/web/search.php?n=1). FMF was reported to be autosomal recessive; mutations in pyrin are thought to result in loss of its ability to inhibit inflammasomes. Nowadays, pyrin assembles with ASC and pro-caspase-1 to form the pyrin inflammasome, as well as the NLRP3 inflammasome [111]. Usually, pyrin is phosphorylated by serine/threonine-protein kinases PKN1 and PKN2, and inhibited by 14-3-3 proteins. When virulence factors expressed or secreted by bacteria and/or viruses inhibit RhoA GTPase, the pyrin inflammasome triggers activation and secretion of IL-1β [112] (Fig. 3). *Yersinia pestis*-like bacteria have a YopM protein which interacts with pyrin to inhibit inflammatory responses for avoiding further anti-bacterial responses [113]. In patients with FMF, pyrin harboring mutant human B30.2 domains defect such kind of ability, thereby preventing binding to ASC; this makes prolonged inflammasome activation and IL-1β secretion [114] (Fig. 3). Corresponding common diseases caused by the similar signaling are shown in Table 1.

### Periodic fever immunodeficiency and thrombocytopenia

**Fig. 4:**
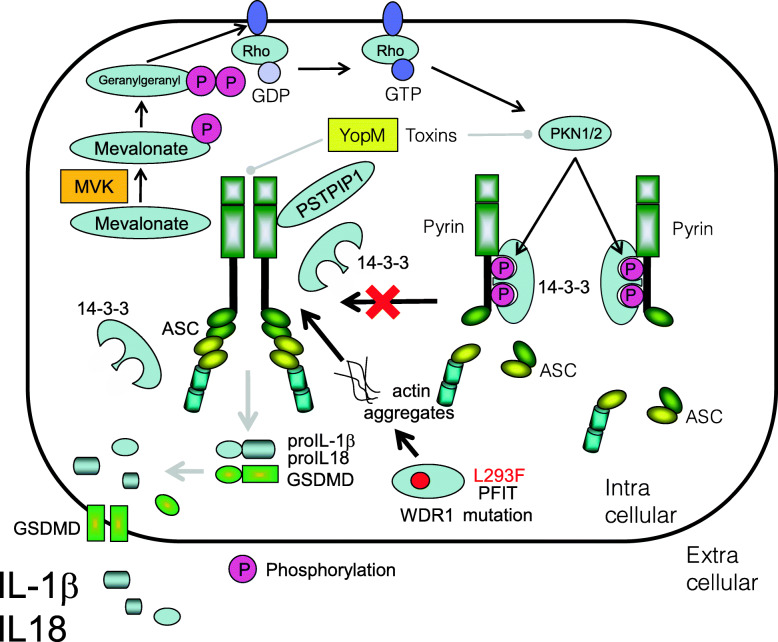
Activation of pyrin inflammasome is inhibited by phosphorylation by PKN1/2. Upon bacterial infection, bacterial toxin can inhibit PKN1/2 activity, resulting in pyrin inflammasome activation. The L293F mutation in WDR1 leads to actin accumulation and aggregates with pyrin, resulting in pyrin activation

The causative gene product of periodic fever immunodeficiency and thrombocytopenia (PFIT) is WDR1 [115, 116], which interacts with cofilin to promote cleavage and depolymerization of F-actin [117, 118]. The L293F mutation in WDR1 disrupts intramolecular hydrophobic interactions, which are important for maintaining actin protein structure. This disruption leads to actin accumulation and aggregates with pyrin resulting in pyrin activation and release of IL-18 [119] (Fig. 4). Corresponding common diseases caused by the similar signaling are shown in Table 1.

### Pyrin-associated autoinflammation with neutrophilic dermatosis

**Fig. 5:**
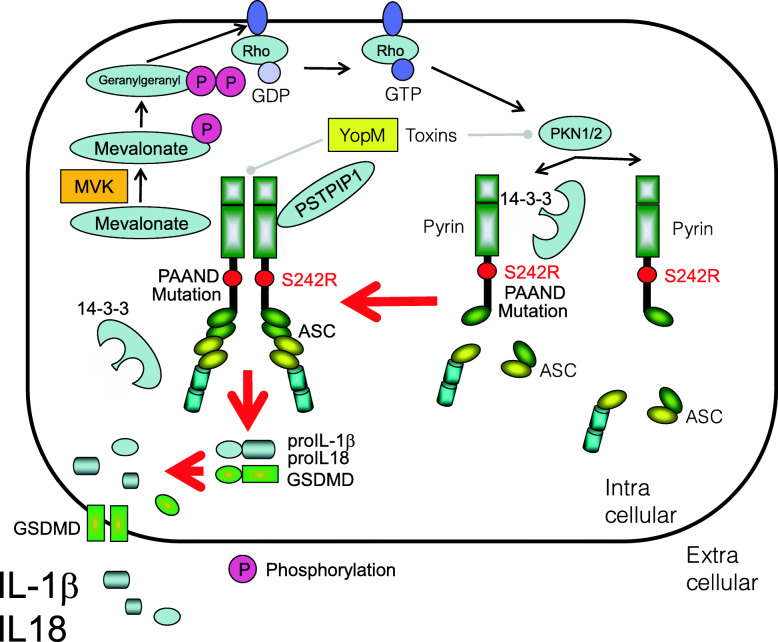
Activation of pyrin inflammasome in patients with pyrin-associated autoinflammation with neutrophilic dermatosis (PAAND). Pyrin mutations in patients with PAAND (e.g., S242R and E244K) mutations are located in the 14-3-3 binding motif, which interferes with the binding of pyrin to 14-3-3, thereby allowing activation of the pyrin inflammasome

The *MEFV* mutations in patients with pyrin-associated autoinflammation with neutrophilic dermatosis (PAAND) harbor S242R and E244K mutations in pyrin; these mutations are located in the 14-3-3 binding motif, which interferes with binding of pyrin to 14-3-3, thereby allowing assembly of the pyrin inflammasome and excessive release of IL-1β [120–123] (Fig. 5). Corresponding common diseases caused by the similar signaling are shown in Table 1.

### Pyogenic arthritis, pyoderma gangrenosum, and acne syndrome

**Fig. 6:**
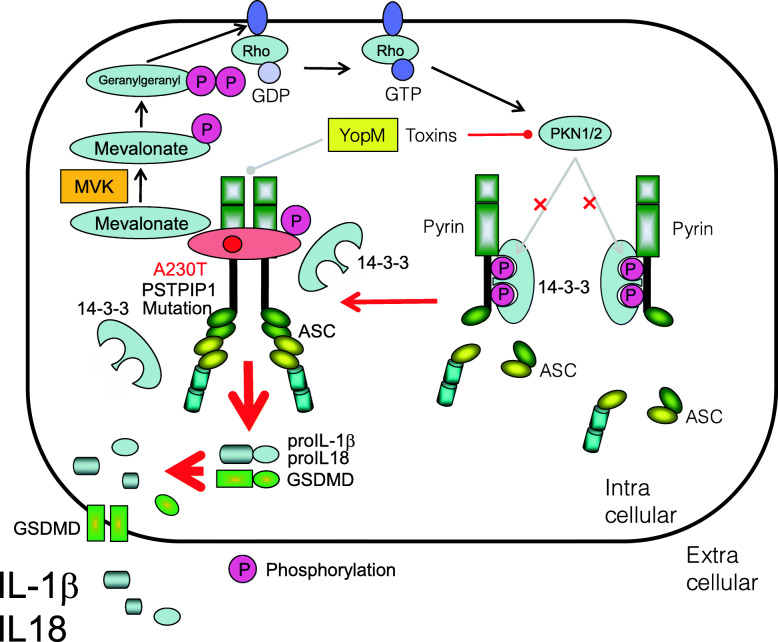
Activation of pyrin inflammasome in patients with pyogenic arthritis, pyoderma gangrenosum, and acne (PAPA) syndrome. Mutations of PSTPIP1 in patients with PAPA (e.g., A230T and E250Q) result in hyperphosphorylation of PSTPIP1, which strengthens its interaction with pyrin via the B-box domain to activate the pyrin inflammasome

The causative gene product of pyogenic arthritis, pyoderma gangrenosum, and acne (PAPA) syndrome is proline-serine-threonine phosphatase-interacting protein 1 (PSTPIP1) (also called CD2-binding protein 1 (CD2BP1)) [124, 125]. Currently, 66 variants of the *PSTPIP1* gene have been reported (https://infevers.umai-montpellier.fr/web/search.php?n=5). In patients with PAPA syndrome, mutations in PSTPIP1 result in hyperphosphorylation of PSTPIP1, which strengthens its interaction with pyrin via the B-box domain to activate the pyrin inflammasome. This leads to increased secretion of IL-1β [125] (Fig. 6). Corresponding common diseases caused by the similar signaling are shown in Table 1.

### Mevalonate kinase deficiency/hyper-IgD syndrome

**Fig. 7:**
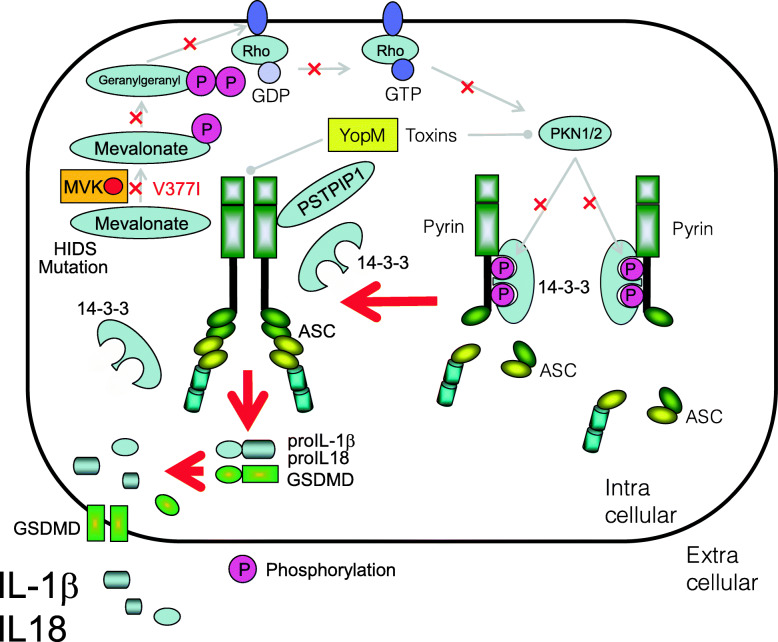
Activation of pyrin inflammasome in patients with hyper-IgD syndrome (HIDS). Deficiency of MVK activity by loss-of-function mutations leads to depletion of geranylgeranyl pyrophosphate, resulting in the inactivation of RhoA and following activation of PKN1/2. This causes activation of pyrin inflammasome

The causative gene product of mevalonate kinase deficiency/hyper-IgD syndrome (MKD) (also known as hyper-IgD syndrome (HIDS)) is mevalonate kinase (MVK) [126]. Currently, 264 variants of this gene have been reported (https://infevers.umai-montpellier.fr/web/search.php?n=3). Geranylgeranyl pyrophosphate, the substrate of geranylgeranylation, is a product of the mevalonate pathway. Deficiency of MVK leads to depletion of geranylgeranyl pyrophosphate, resulting in the inactivation of RhoA [127, 128]. Since the inactivation of RhoA activates the pyrin inflammasome, MKD leads to an inflammasomopathy. Indeed, canakinumab, an anti-IL-1β monoclonal antibody, is an effective treatment for MKD, suggesting that IL-1β is a common mediator of these diseases [129] (Fig. 7). Corresponding common diseases caused by the similar signaling are shown in Table 1.

### NLRC4 inflammasomopathies

**Fig. 8:**
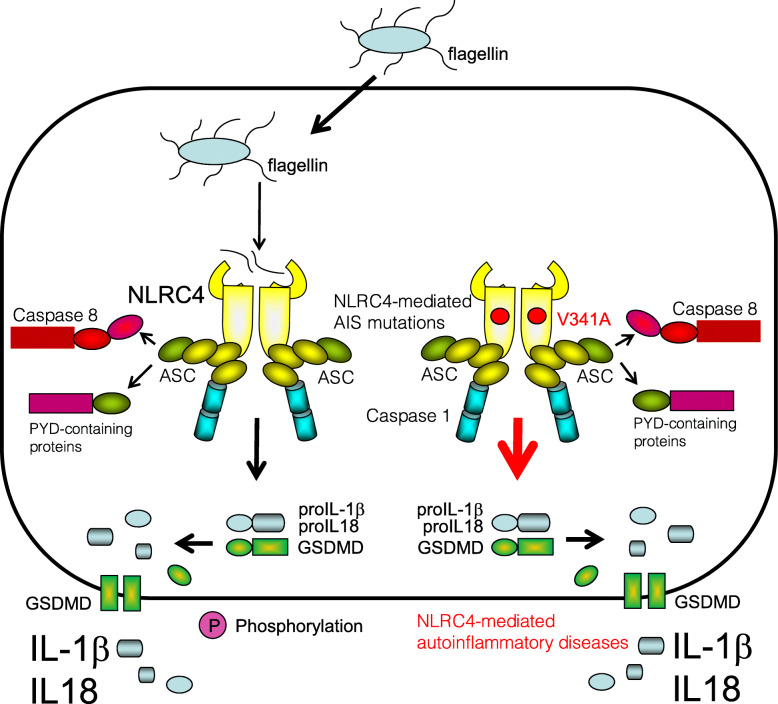
NLRC4 inflammasomopathies are related to gain-of-function mutations of NLRC4 inflammasome. Gain-of-function of mutations in NLRC4 in patients with NLRC4 inflammasomopathies constitutively actives for NLRC4 inflammasome. The NLRC4 inflammasome activates caspase-1 either with or without an adaptor ASC leading to hypersecretion of IL-18 and IL-1β

Gain-of-function mutations in NLRC4 result in early-onset recurrent fever and macrophage activation syndrome (MAS), neonatal-onset enterocolitis with periodic fever, fatal or near-fatal episodes of autoinflammation, or symptoms resembling those of FCAS [68, 130, 131]. So far, more than 31 genetic variants of NLRC4 have been reported (https://infevers.umai-montpellier.fr/web/search.php?n=25). The NLRC4 inflammasome activates caspase-1 either with or without an adaptor ASC, which in turn activates IL-1β and IL-18. NLRC4 inflammasomopathies are linked more closely with hypersecretion of IL-18 rather than of IL-1β; however, the precise mechanism remains to be elucidated [132] (Fig. 8). Corresponding common diseases caused by the similar signaling are shown in Table 1.

## NF-κB-related autoinflammatory diseases (relopathies)

**Fig. 9:**
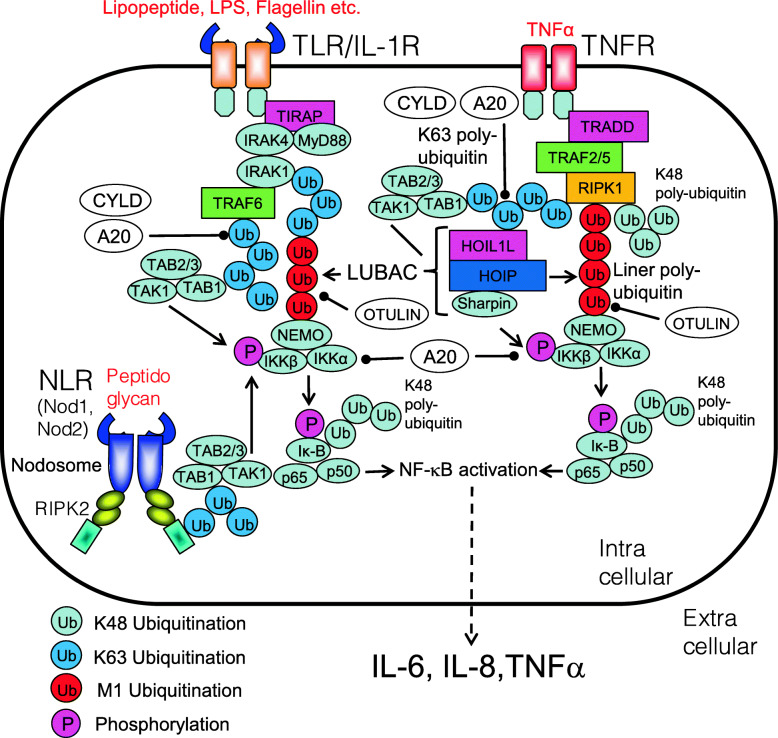
Autoinflammatory disease-related NF-κB activation pathway. In normal, upon recognition of DAMPs, PAMPs, or appropriate ligands, TLR, TNFR, Nod1, and Nod2 activate NF-κB. Dysregulations of the NF-κB signaling are closely linking to autoinflammatory diseases called relopathies. The NF-κB activation pathway is regulated by polyubiquitination chains

Dysregulations of NF-κB signaling are closely linking to the ubiquitination system. In addition to constitutive activation of NF-κB, loss-of-function mutations in the ubiquitin-mediated NF-κB regulatory system cause autoinflammatory diseases [10] (Fig. 9).

### Blau syndrome/early-onset sarcoidosis

**Fig. 10:**
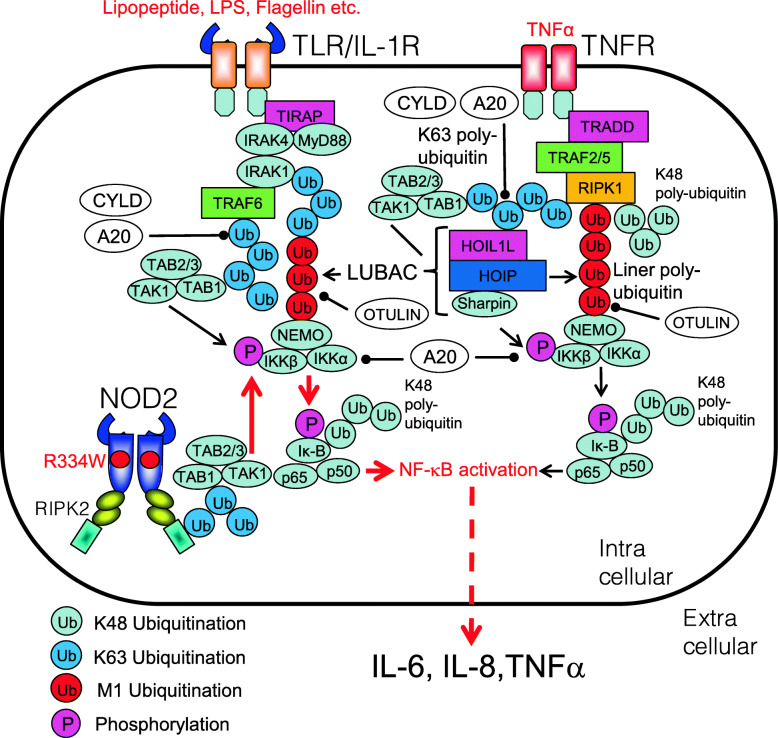
NF-κB activation pathway in patients with Blau syndrome (BS)/early-onset sarcoidosis (EOS). NOD2 recognizes muramyl dipeptide (MDP), a minimum component of peptidoglycan, leading to activation of NF-κB. Gain-of-function mutations in NOD2 (e.g., R334W) increase signaling via NOD2-RIPK2-associated activation of NF-κB

The gene responsible for Blau syndrome (BS)/early-onset sarcoidosis (EOS) is *IBD1*, and its causative gene product is NOD2 [133]. Usually, NOD2 recognizes muramyl dipeptide (MDP), leading to activation of NF-κB. Currently, 185 variants of NOD2 have been reported (https://infevers.umai-montpellier.fr/web/search.php?n=6). Gain-of-function mutations in NOD2 increase signaling via NOD2-RIPK2-associated activation of NF-κB [134, 135] (Fig. 10). Corresponding common diseases caused by the similar signaling are shown in Table 1.

### A20 protein haploinsufficiency

**Fig. 11:**
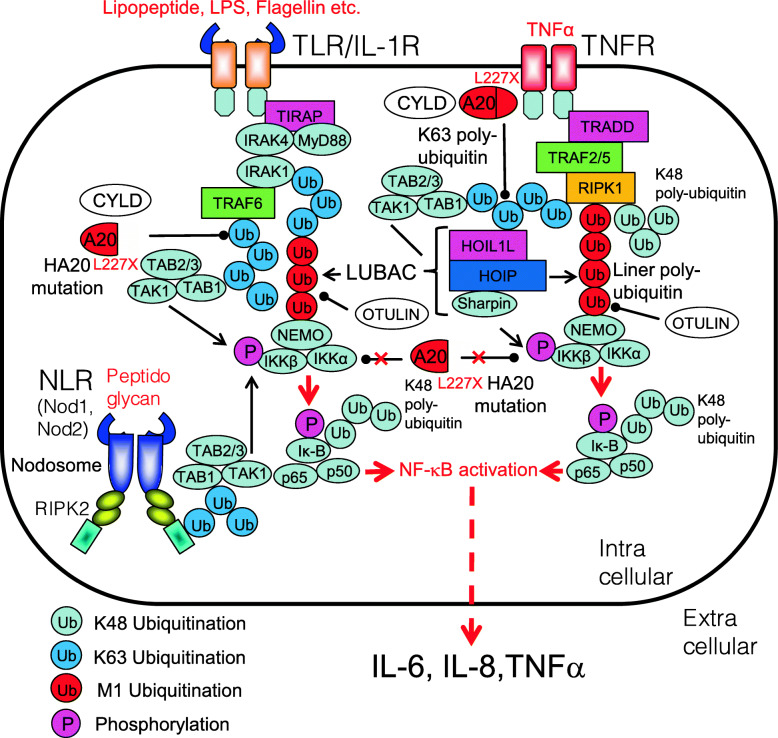
NF-κB activation pathway in patients with A20 protein haploinsufficiency (HA20). Loss-of-function mutations of A20 (e.g., L227X) reduce the deubiquitination activity of A20 leading to prolonged activation of NF-κB

A20 (also called TNF-α-induced protein (TNFAIP) 3, is an intracellular deubiquitinase. A20 plays a role in deubiquitination of several proteins, including NF-κB. A20 protein haploinsufficiency (HA20) is caused by heterozygous mutation or deletion of A20, resulting in insufficient deubiquitination of TRAF6 downstream of the TNF-α pathway, RIPK1 downstream of the TLR pathway, and RIPK2 downstream of the NOD1 or NOD2 pathways. Loss of A20 function leads to constitutive activation of NF-κB signaling [136, 137]. A20 also regulates the activity of the NLRP3 inflammasome in macrophages [138]. So far, 55 variants of A20 have been reported (https://infevers.umai-montpellier.fr/web/search.php?n=26). Haplodeficient mutations severely reduce A20 function, leading to prolonged activation of NF-κB [139] (Fig. 11). Corresponding common diseases caused by the similar signaling are shown in Table 1.

### Immunodeficiency, autoinflammation, and amylopectinosis with inherited linear ubiquitin chain assembly complex deficiency

**Fig. 12:**
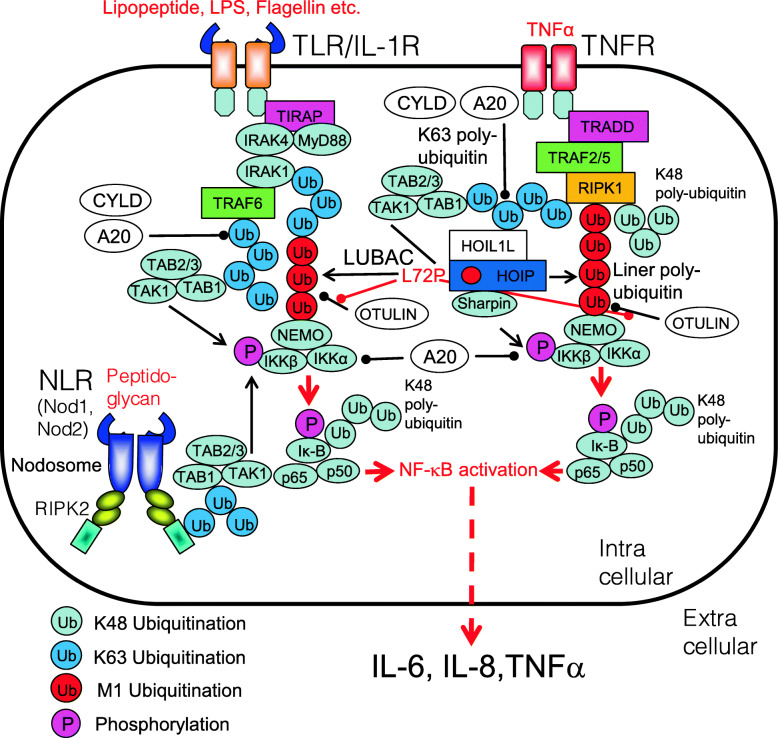
Loss-of-function mutation in linear ubiquitin chain assembly complex (LUBAC) is associated with autoinflammation. Loss-of-function mutations in the HOIP (e.g., L72P) affects its interaction with OTU deubiquitinase with linear linkage specificity (OTULIN) and lysine 63 deubiquitinase (CYLD), leading to prolonged NF-κB activation

Loss-of-function mutation in linear ubiquitin chain assembly complex (LUBAC), a protein complex comprising heme-oxidized IRP2 ubiquitin ligase 1 (HOIL-1) (also called RBCK1), HOIL-1 interaction protein (HOIP, also called RNF31), and SHANK-associated RH domain-interacting protein (SHARPIN) is associated with autoinflammation [140–145]. The L72P mutation in the HOIP protein affects its interaction with OTU deubiquitinase with linear linkage specificity (OTULIN) and lysine 63 deubiquitinase (CYLD); however, the most common disease-causing phenomenon is loss of expression of the L72P allele of HOIP. Combined heteromutations comprise L41fsX7 and Q185X, which result in deficient HOIL-1 expression. Lack of HOIL-1 expression by fibroblasts impairs phosphorylation of IKK kinase, slower degradation of IκBα, and decreased ubiquitination of NEMO in response to stimulation with either TNF-α or IL-1β. LUBAC deficiency in fibroblasts downregulates NF-κB activation in response to IL-1β or TNF-α, whereas deficient monocytes release more IL-6 but less IL-10 in response to IL-1β [146–148] (Fig. 12). Corresponding common diseases caused by the similar signaling are shown in Table 1.

### OTULIN-related autoinflammatory syndrome

**Fig. 13:**
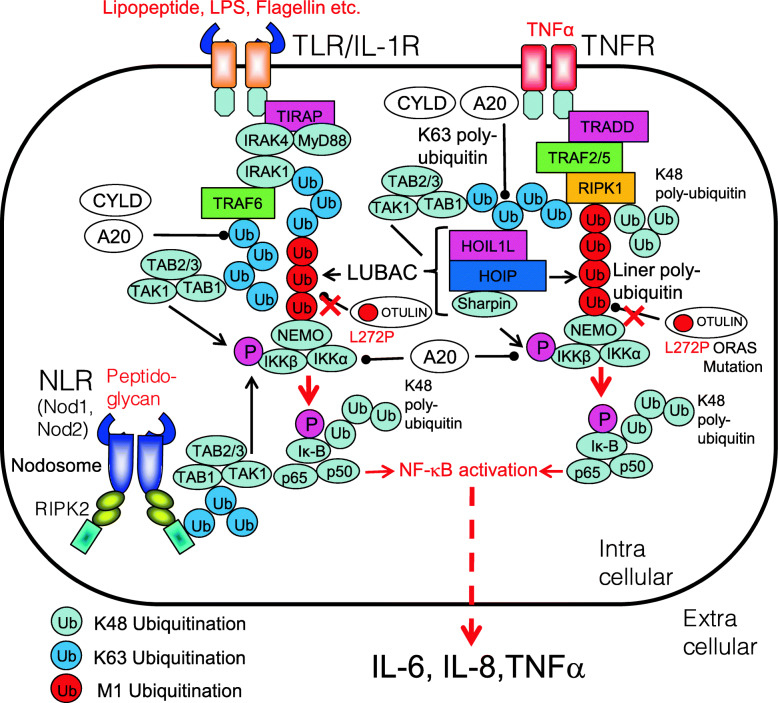
Homozygous loss-of-function mutations in OTULIN in patients with OTULIN-related autoinflammatory syndrome (ORAS). OTULIN is a deubiquitination enzyme that hydrolyzes methionine-1 (M1), which links to liner ubiquitin chains to regulate the activity of NF-κB. The L272P mutation of OTULIN disrupts the binding of OTULIN and ubiquitin to its substrate, leading to prolonged NF-κB activation

OTULIN is a deubiquitination enzyme that hydrolyzes methionine-1 (M1), which links to liner ubiquitin chains to regulate the activity of NF-κB [149]. Homozygous loss-of-function mutations in OTULIN cause OTULIN-related autoinflammatory syndrome (ORAS) [150]. The L272P mutation is located in a helix of the catalytic OTU domain, which forms part of the binding pocket for M1-linked distal ubiquitin; this mutation disrupts the binding of OTULIN and ubiquitin to its substrate [151, 152] (Fig. 13). Corresponding common diseases caused by the similar signaling are shown in Table 1.

## IL-1 receptor-related autoinflammatory diseases

IL-1 receptor-related autosomal recessive autoinflammatory diseases are caused by mutations in IL1RN (interleukin-1 receptor antagonist), resulting in a condition called deficiency of interleukin-1 receptor antagonist (DIRA) [153–155]. So far, 22 variants of this gene have been reported (https://infevers.umai-montpellier.fr/web/search.php?n=10). IL-1RA deficiency results in uncontrolled IL-1α, IL-1β and NF-κB signaling [156] (Fig. 9). Corresponding common diseases caused by the similar signaling are shown in Table 1.

## Interferonopathies

**Fig. 14:**
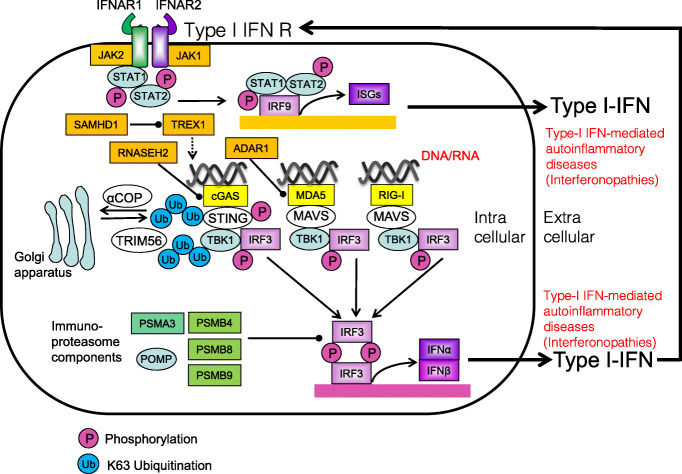
Type I interferon signaling in interferonopathies. Type I interferon singling is closely linked to innate immune receptors such as cGAS, MDA5, and RIG-I that are sensing viral, bacterial, or own DNA or RNA. Interferon-stimulated genes (ISGs) are induced by interferon regulatory factors (IRFs) downstream of cGAS, MDA5, and RIG-I. Immunoproteasome dysfunction is also linked to interferonopathies

Anti-viral first-line defense is dependent on innate immune receptors (e.g., cGAS, MDA5, and RIG-I) that are detecting intracellular viral, bacterial, or own nucleic acid, linking to type I interferon signaling. Interferonopathies are closely linked to dysfunction of these innate immune receptors and type I interferon signaling, Immunoproteasome dysfunction is also linked to the interferonopathies [157] (Fig. 14).

### Aicardi–Goutières syndrome

**Fig. 15:**
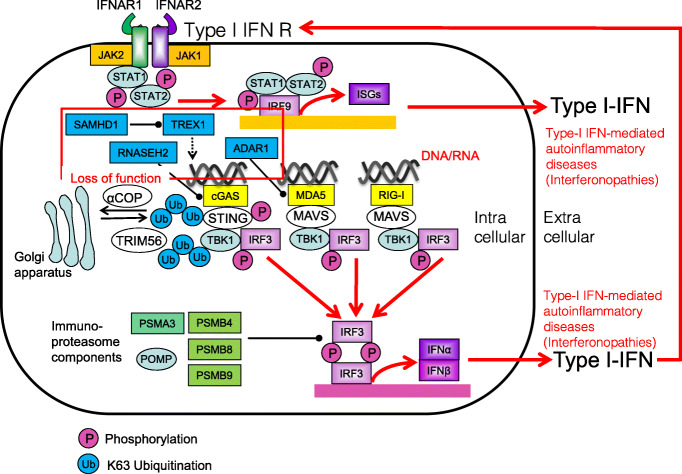
Loss-of-function mutations in TREX1, RNASEH2A, RNASEH2B, RNASEH2C, SAMHD1, or ADAR1 in patients with Aicardi–Goutières syndrome (AGS). Loss-of-function mutations in TREX1, RNASEH2A, RNASEH2B, RNASEH2C, SAMHD1, or ADAR1 results in the accumulation of nucleic acids which leads to the induction of IRF transcription

Aicardi–Goutières syndrome (AGS) is an inherited encephalopathy that affects newborn infants and usually results in severe neuro-physical disability. AGS is caused by loss-of-function mutations in the genes encoding the three prime repair exonuclease 1 (TREX1), the ribonuclease H2 subunit (RNASEH2)A, RNASEH2B, RNASEH2C, the phosphohydrolase SAM domain and HD domain-containing protein 1 (SAMHD1), or the dsRNA-specific adenosine deaminases acting on RNA1 (ADAR1) [158, 159]. In addition, gain-of-function mutations in the dsRNA sensor MDA5 (also called IFIH1) have been identified in AGS patients [157]. AGS pathology seems to be caused by the accumulation of nucleic acids, which can cause neurological and liver abnormalities that resemble congenital viral infection (Fig. 15). Corresponding common diseases caused by the similar signaling are shown in Table 1.

### Stimulator of interferon gene-associated vasculopathy with onset in infancy

Stimulator of interferon gene (STING)-associated vasculopathy with onset in infancy (SAVI) is caused by gain-of-function mutations in STING (also called TMEM173). Mutation of the STING amplifies the function of STING, which is an adaptor molecule involved in signal transduction through cGAS, leading to hyperactivation of type I IFN pathways [160]. Corresponding common diseases caused by the similar signaling are shown in Table 1.

### Coatomer protein alpha syndromes

**Fig. 16:**
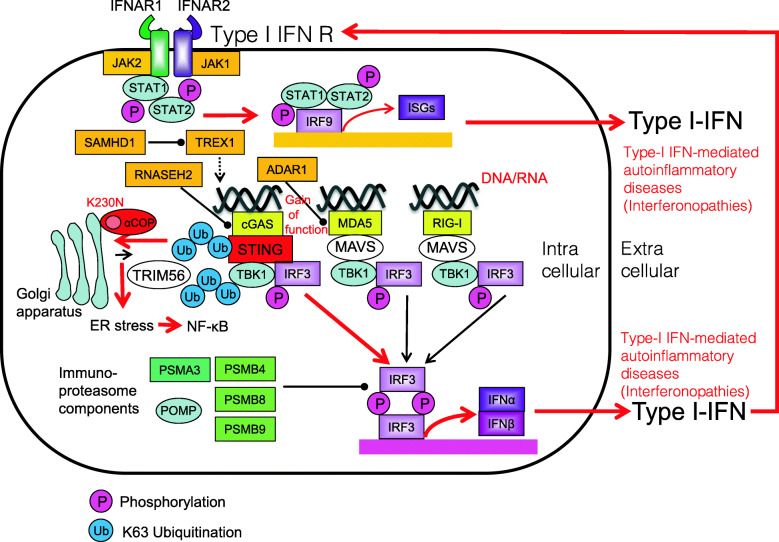
Mutations in αCOP in patients with COPA syndrome. Mutations in αCOP (e.g., K230N) impair ER-Golgi transport resulting in ER stress-induced NF-κB activation and the mutant αCOP retention of STING on the Golgi resulting in STING activation leads to prolonged type I interferon signaling

Coatomer protein alpha (COPA) syndrome, characterized by high-titer autoantibodies, interstitial lung disease, and inflammatory arthritis, was found to be deleterious mutations in the COPA gene (encoding coatomer subunit α). Mutant COPA causes defective intracellular transport via coat protein complex I which leads to ER stress and the upregulation of the levels of transcripts encoding IL-1β, IL-6, and IL-23 [161]. COPA is a critical regulator of STING transport ER and retrieval of STING from the Golgi. Mutant COPA retention of STING on the Golgi resulting in STING activation leads to prolonged type I interferon signaling [86] (Fig. 16). Corresponding common diseases caused by the similar signaling are shown in Table 1.

### Proteasome-associated autoinflammatory syndromes

**Fig. 17:**
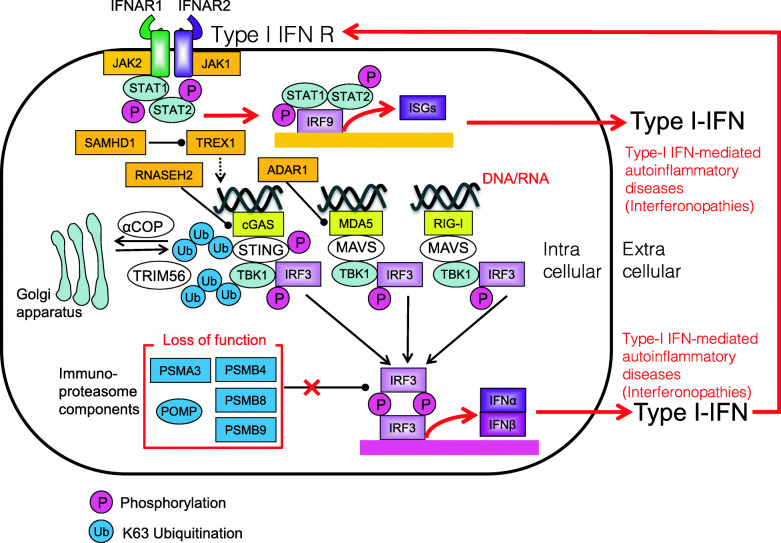
Loss-of-function mutations in immunoproteasome components PSMB8, PSMB4, PSMA3, PSMB9, or proteasome maturation protein (POMP) in patients with PRAAS/NNS/CANDLE. Loss-of-function mutation in immunoproteasome components such as PSMB8, PSMB4, PSMA3, PSMB9, or proteasome maturation protein (POMP) leads to increased secretion of type I IFN by immune cells

Nakajo–Nishimura syndrome (NNS) and chronic atypical neutrophilic dermatosis with lipodystrophy and elevated temperature syndrome (CANDLE) were the first PRAAS to be described. Loss-of-function mutation in immunoproteasome components such as proteasome subunit beta type (PSMB)8, PSMB4, PSMA3, PSMB9, or proteasome maturation protein (POMP) leads to increased secretion of type I IFN by immune cells [162, 163] (Fig. 17). Corresponding common diseases caused by the similar signaling are shown in Table 1.

### Singleton–Merten syndrome

**Fig. 18:**
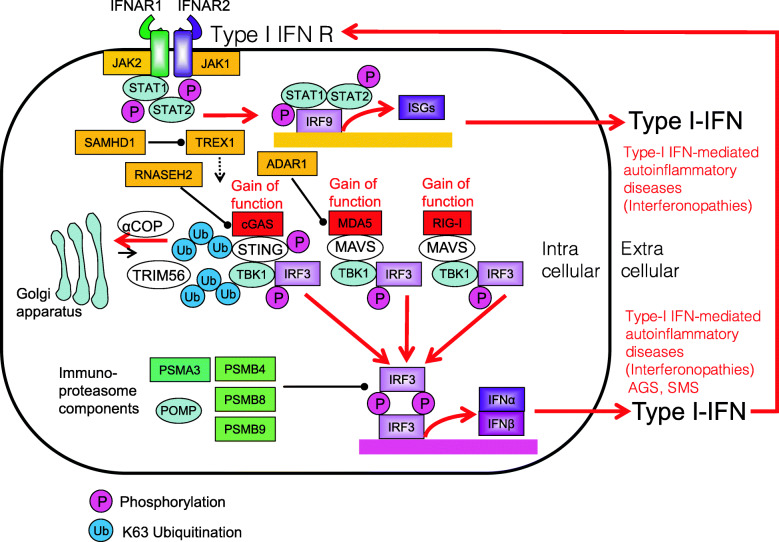
Gain-of-function mutations in MDA5 or RIG-I in patients with Singleton-Merten syndrome (SMS). Typical SMS is caused by gain-of-function mutations in MDA5. Atypical SMS is caused by gain-of-function mutations in RIG-I. Gain-of-function mutations of MDA5 and RIG-I lead to constitutive activation of type I IFN signaling pathways

Singleton–Merten syndrome (SMS) is caused by gain-of-function mutations in the RNA sensor MDA5 or RIG-I. Typical SMS is caused by a mutation in MDA5, whereas atypical SMS is caused by a mutation in RIG-I; both mutations cause constitutive activation of IFN signaling pathways [164, 165]. Notably, mutations in the MDA5 are also associated with AGS, so that both SMS and AGS share a common molecular mechanism [164] (Fig. 18). Corresponding common diseases caused by the similar signaling are shown in Table 1.

## Conclusions

Here, we describe briefly the molecular mechanisms underlying autoinflammatory diseases caused by dysregulation of IL-1β or IL-18 processing, NF-κB activation, and IFN secretion. Disruption of the fine balance within these signaling pathways contributes to the pathogenesis of autoinflammatory diseases. Increasing our knowledge of the molecular biology underlying autoinflammatory diseases will facilitate the development of disease-targeting biologics. Therefore, future studies should elucidate the autoinflammatory disease-specific signalosome in detail.

## Data Availability

Not applicable.

## Abbreviations

DAMP: Damage-associated molecular pattern
PAMP: Pathogen-associated molecular pattern
PRR: Pattern recognition receptor
TLR: Toll-like receptor
NLR: NOD-like receptor
CLR: C-type lectin receptor
RLR: RIG-I-like receptor
NF: Nuclear factor
PRAAS: Proteasome-associated autoinflammatory syndromes
IL: Interleukin
PYD: Pyrin domain
AIM2: Absent in melanoma 2
ASC: Apoptosis-associated speck-like protein containing a caspase-recruitment domain
CARD: Caspase-recruitment domain
GSDMD: Gasdermin D
CAPS: Cryopyrin-associated periodic syndrome
FCAS: Familial cold autoinflammatory syndrome
FCU: Familial cold urticaria
MWS: Muckle–Wells syndrome
NOMID: Neonatal-onset multisystem inflammatory disease
CINCA: Chronic infantile neurologic, cutaneous, and arthritis
NAIAD: NLRP1-associated autoinflammation with arthritis and dyskeratosis
NLRP12-AD: NLRP12 autoinflammatory syndrome
TNF: Tumor necrosis factor
TRAPS: TNF receptor-associated periodic fever syndrome
TNFRSF: TNF receptor superfamily member
APLAID: Autoinflammation and PLCγ2-associated antibody deficiency and immune dysregulation
FMF: Familial Mediterranean fever
PFIT: Periodic fever immunodeficiency and thrombocytopenia
PAAND: Pyrin-associated autoinflammation with neutrophilic dermatosis
PAPA: Pyogenic arthritis, pyoderma gangrenosum, and acne
PSTPIP1: Proline-serine-threonine phosphatase-interacting protein 1
CD2BP1: CD2-binding protein 1
MKD: Mevalonate kinase deficiency
HIDS: Hyperimmunoglobulinemia D and periodic fever syndrome
MVK: Mevalonate kinase
MAS: Macrophage activation syndrome
BS: Blau syndrome
EOS: Early-onset sarcoidosis
MDP: Muramyl dipeptide
HA20: A20 protein haploinsufficiency
TNFAIP: Tumor necrosis factor alpha-induced protein
IAALUCD: Immunodeficiency, autoinflammation, and amylopectinosis with inherited linear ubiquitin chain assembly complex deficiency
LUBAC: Linear ubiquitin chain assembly complex
HOIL-1: Heme-oxidized IRP2 ubiquitin ligase 1
HOIP: HOIL-1 interaction protein
SHARPIN: SHANK-associated RH domain-interacting protein
OTULIN: OTU deubiquitinase with linear linkage specificity
CYLD: CYLD lysine 63 deubiquitinase
ORAS: OTULIN-related autoinflammatory syndrome
DIRA: Deficiency of the IL-1-receptor antagonist
AGS: Aicardi–Goutières syndrome
TREX: Three prime repair exonuclease
RNASEH2: Ribonuclease H2 subunit
SAMHD: SAM domain and HD domain-containing protein
ADAR1: dsRNA-specific adenosine deaminases acting on RNA 1
STING: Stimulator of interferon genes
SAVI: STING-associated vasculopathy with onset in infancy
COPA: Coatomer protein alpha
NNS: Nakajo–Nishimura syndrome
CANDLE: Chronic atypical neutrophilic dermatosis with lipodystrophy and elevated temperature syndrome
PSMB: Proteasome subunit beta type
POMP: Proteasome maturation protein
SMS: Singleton–Merten syndrome
